# Time Course of Redox Biomarkers in COVID-19 Pneumonia: Relation with Inflammatory, Multiorgan Impairment Biomarkers and CT Findings

**DOI:** 10.3390/antiox10071126

**Published:** 2021-07-14

**Authors:** Tijana Kosanovic, Dragan Sagic, Vladimir Djukic, Marija Pljesa-Ercegovac, Ana Savic-Radojevic, Zoran Bukumiric, Miodrag Lalosevic, Marjana Djordjevic, Vesna Coric, Tatjana Simic

**Affiliations:** 1 Radiology Department, The University Hospital ‘Dr. Dragisa Misovic- Dedinje’, 11000 Belgrade, Serbia; tijana.kosanovic@dragisamisovic.bg.ac.rs (T.K.); office@dragisamisovic.bg.ac.rs (V.D.); miodrag.lalosevic@dragisamisovic.bg.ac.rs (M.L.); marjana.djordjevic@dragisamisovic.bg.ac.rs (M.D.); 2 Institute for Cardiovascular Diseases “Dedinje”, 11000 Belgrade, Serbia; dragan.sagic@med.bg.ac.rs; 3 Institute of Medical and Clinical Biochemistry, Faculty of Medicine, University of Belgrade, 11000 Belgrade, Serbia; marija.pljesa-ercegovac@med.bg.ac.rs (M.P.-E.); ana.savic-radojevic@med.bg.ac.rs (A.S.-R.); 4 Institute of Medical Statistics and Informatics, School of Medicine, University of Belgrade, 11000 Belgrade, Serbia; zoran.bukumiric@med.bg.ac.rs; 5 Serbian Academy of Science and Arts, 11000 Belgrade, Serbia

**Keywords:** COVID-19, oxidative stress, MDA, AOPP, 8-OHdG, CT pulmonary patterns

## Abstract

Although the original data on systemic oxidative stress in COVID-19 patients have recently started to emerge, we are still far from a complete profile of changes in patients’ redox homeostasis. We aimed to assess the extent of oxidative damage of proteins, lipids and DNA during the course of acute disease, as well as their association with CT pulmonary patterns. In order to obtain more insight into the origin of the systemic oxidative stress, the observed parameters were correlated with inflammatory biomarkers and biomarkers of multiorgan impairment. In this prospective study, we included 58 patients admitted between July and October 2020 with COVID-19 pneumonia. Significant changes in malondialdehyde, 8-hydroxy-2’-deoxyguanosine and advanced oxidation protein products levels exist during the course of COVID-19. Special emphasis should be placed on the fact that the pattern of changes differs between non-hospitalized and hospitalized individuals. Our results point to the time-dependent relation of oxidative stress parameters with inflammatory and multiorgan impairment biomarkers, as well as pulmonary patterns in COVID-19 pneumonia patients. Correlation between redox biomarkers and immunological or multiorgan impairment biomarkers, as well as pulmonary CT pattern, confirms the suggested involvement of neutrophils networks, IL-6 production, along with different organ/tissue involvement in systemic oxidative stress in COVID-19.

## 1. Introduction

Efforts to decipher the pathophysiology of acute respiratory syndrome (SARS) coronavirus 2 (SARS-CoV-2) infection, from the very beginning of the COVID-19 pandemic challenge, have been pointing to the pleiotropic roles of free radicals, including the interaction of the virus with its receptor (i), viral replication (ii), interplay of cytokine storm and free radical storm in overactive immune response induced by virus (iii) and consequent end-organ damage (iv) [1,2,3,4]. Although the original data on systemic oxidative stress in COVID-19 patients have recently started to emerge, we are still far from a complete profile of changes in patients’ redox homeostasis, similar to, e.g., well-defined changes in cytokines and chemokines production [5]. So far, data obtained in clinical settings suggest enormous consumption of exogenous antioxidants, since the levels of vitamin C are undetectable in patients admitted to intensive care [6], while alpha tocopherol and beta carotene concentrations are also decreased [7] followed by disrupted endogenous thiol disulphide homeostasis in COVID-19 [8,9,10]. Data on lipid oxidation in COVID patients have been controversial, since increased plasma levels of isoprostanes F2 alpha, followed by decreased malondialdehyde (MDA) levels, were shown in comparison with controls [11]. 

Stratification of COVID-19 disease severity, ranging from asymptomatic or mild to moderate, severe or critical, has been well established over the course of the pandemic. Thus, patients with mild disease symptoms exhibit rapid recovery, while those with moderate to severe disease exhibit high mortality and poor prognosis [12]. It has been suggested that in addition to alterations of well-recognized immunological mechanisms, endothelial dysfunction induced by impaired hypoxia-related redox signaling also contributes to SARS-CoV-2 pathogenicity [13]. Still, previous evidence that severity and progression in hospitalized patients with COVID-19 may be related to the systemic changes in their redox profile is conflicting and requires further investigation [7,9,11,14,15]. Besides, the majority of clinical studies were designed to determine the level of redox biomarkers in COVID-19 patients at one point in time, without taking into account the time course of the oxidative damage during disease progression. In view of the fact that evaluation of systemic oxidative stress in humans relies on determination of byproducts reflecting oxidative damage of different macromolecules, it is important to assess both protein and DNA oxidative modifications, e.g., advanced oxidation protein products (AOPP) and 8-hydroxy-2’-deoxyguanosine (8-OHdG), in addition to lipid damage. It is of particular interest to establish the link of these biomarkers with inflammatory parameters, indices of multiorgan impairment, as well as with severity and progression of pulmonary changes. Namely, the information on redox parameters in COVID-19 pneumonia patients may help us to better understand the natural course of COVID-19 and improve clinical decision making. 

Therefore, we hypothesized that in the acute course of the COVID-19 disease, redox biomarkers correlate with inflammatory and multiorgan impairment biomarkers and are associated with changes in the chest multidetector computed tomography (MDCT) findings in patients with COVID-19 pneumonia. In addition, we aimed to assess if the redox biomarker profile differs between hospitalized patients in comparison with outpatients.

## 2. Materials and Methods

This prospective study included a total of 58 patients with COVID-19 pneumonia, admitted to one of the leading COVID-19 hospitals, the University Hospital ’Dr. Dragiša Mišović-Dedinje’ between July and October 2020, during the second wave of the epidemic in Serbia. All recruited patients had their nasopharyngeal and oropharyngeal swabs taken according to World Health Organization guidelines [16]. All patients had the SARS-CoV-2 PCR detection performed and results issued by official state laboratories. A detailed insight into the genomic characterization of SARS-CoV-2 whole genome sequences from the second wave of the epidemic in Serbia, taking place from June 2020, was reported in the recent study by Miljanovic et al. [17]. Patients were stratified according to hospitalization, comprising those who met the criteria for Stage 3 or higher (according to the COVID-19 National Guidelines, version 9) who were hospitalized (n = 42) and the remaining 16 outpatients. Twenty-two inpatients required oxygenation and none of them required intubation or had acute respiratory distress syndrome—ARDS.

The principles of ICH Good Clinical Practice, the ‘Declaration of Helsinki’ and national and international ethical guidelines were strictly followed during this study. Approval from the Ethics Committee in The University Hospital ’Dr. Dragiša Mišović-Dedinje’, Belgrade, Serbia was obtained (No01-7661, date 1 July 2020). Informed written consent was procured from all recruited subjects.

All patients had their laboratory analysis and non-contrast chest CT estimated on the day of admission, on the 7th and on the 14th day, consecutively. All CT scans were acquired using the Canon (former Toshiba), Aquillion One (TSX-301C), 320 row MDCT System (Canon, Tokyo, Japan). Scans acquisition was conducted from the level of the thoracic entrance to the inferior level of the costophrenic angle, with the patient in a supine, arms raised, head forward position and with breath-holding manner during end inspiration. Unenhanced CT scans were obtained for all patients. The following parameters were used: tube voltage 120 kV with automatic tube current modulation, slice thickness 1.0 mm. All CT images were reviewed by two experienced radiologists with extensive experience in thoracic imaging on a diagnostic workstation (Vitrea extend-Vital, Canon, Tokyo, Japan) with multiplanar reconstruction (MPR) tools. All disagreements between them were resolved by consensus of acceptance by both radiologists. The images were viewed in the lung window settings (width, 1600 HU; level, 400 HU) and mediastinal (soft tissue) window settings of width, 380 HU; level, 40 HU. Assessed CT pulmonary patterns comprised ground glass opacities (GGO), crazy paving (CP), consolidation (CON), residual ground glass opacities (rGGO), both individual or in respective combination.

EDTA blood was collected for plasma separation. All recruited patients had their blood drawn by venipuncture on admission, 7 and 14 days upon admission. Immediately upon venipuncture, the samples were centrifuged and the obtained plasma aliquoted, frozen and kept at −80 °C until analysis. Malondialdehyde (MDA) was determined in plasma by ELISA kit (Elabscience, E-EL-0060) and 8-hydroxy-2’-deoxyguanosine (8-OHdG) by OxiSelect™ Oxidative DNA Damage ELISA Kit (CellBiolabs, STA-320), whereas advanced oxidation protein products (AOPP) were measured by colorimetric OxiSelect™ AOPP Assay Kit (CellBiolabs, STA-318).

Depending on the type of variables and the normality of the distribution, results were presented as frequency (percent), median (range) and mean ± standard deviation. Statistical hypotheses were tested using ANOVA with repeated measures, Friedman test or Wilcoxon test. The correlation between the variables was estimated using Spearman’s correlation coefficient (rho). Changes in parameters (MDA, 8-OHdG and AOPP) were compared between hospitalized and non-hospitalized patients across time (during the course of the study: on admission, 7 and 14 days upon admission) with linear mixed effects modeling approach using R package nlme version 3.1-147. Regarding the models fit, it was assessed using Akaike’s information criterion (AIC); AIC value is 2276.3 for MDA model, 757.0 for 8-OHdG and 2115.5 for AOPP model. Results were graphically presented using the R package ggplot2 version 3.3.2. Statistical hypotheses were analyzed at the level of significance of 0.05. Statistical data analysis was performed using IBM SPSS Statistics 22 (IBM Corporation, Armonk, NY, USA) and R-3.5.0 software (The R Foundation for Statistical Computing, Vienna, Austria).

## 3. Results

The study group comprised a total of 58 patients (Table 1) with COVID-19 pneumonia, admitted 4–6 days upon symptoms onset. The majority of patients were hospitalized (n = 42, 72%). Most patients exhibited symptoms including fever (72%), tiredness (59%) and cough (45%). Myalgia, dyspnea and diarrhea were observed in less than 30% of patients. The group comprised only one smoker and eight patients previously diagnosed with regulated hypertension, with an average cohort BMI 28.03 ± 5.34 kg/m^2^ (Table 1). The therapy that was administered over the course of the disease is presented in Table 2. As indicated, all patients received antioxidant supplementation and the majority received anticoagulant therapy. On the other hand, the remaining therapy was administered in less than 30%, being quite heterogeneous.

### 3.1. Time Course of Redox Biomarkers in Patients with COVID-19 Pneumonia

The change across all three time points was significant for all inflammatory biomarkers, multiorgan impairment biomarkers and redox biomarkers in patients with COVID-19 pneumonia (*p* < 0.05, Table 3). Additionally, the overview of the inflammatory parameters, multiorgan impairment biomarkers and redox biomarkers in 42 hospitalized as well as 16 non-hospitalized patients with COVID-19 pneumonia, on admission, 7 and 14 days upon admission, are provided in the Tables S1 and S2, respectively (provided).

**Figure 1:**
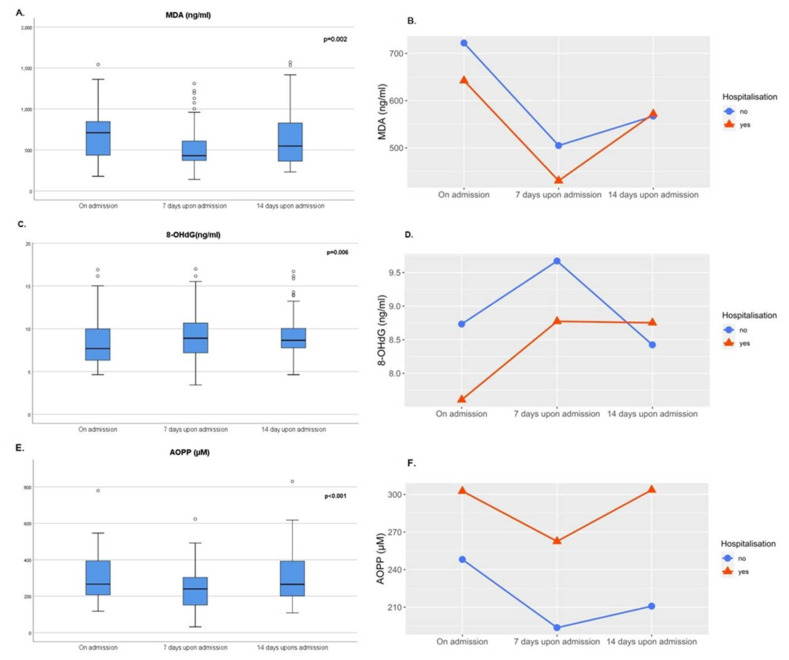
The time course of total (**A**,**C**,**E**) and individual redox biomarkers (MDA, 8-OHdG, AOPP) in hospitalized and non-hospitalized patients with COVID-19 pneumonia (**B**,**D**,**F**); MDA—malondialdehyde; 8-OHdG—8-hydroxy-2’-deoxyguanosine; AOPP—advanced oxidation protein products; *p*-value.

The individual profiles of redox biomarkers (MDA, 8-OHdG, AOPP) for each patient, as well as for each hospitalized patient and non-hospitalized patient are shown in Figures S1 and S2 (provided). The highest median levels of MDA and AOPP were observed at the time of diagnosis (Table 3, Figure 1A,E).

Seven days upon admission, a significant drop in MDA and AOPP levels was observed (*p* < 0.001, Figure 1A,E), while plasma concentration of 8-OHdG increased (*p* = 0.008, Figure 1C). Fourteen days after admission, a noticeable increase was observed in MDA levels (*p* = 0.030) and AOPP (*p* = 0.006), compared to the second time point (Figure 1A,E), while 8-OHdG slightly dropped (*p* > 0.05). After being dichotomized into patients with COVID-19 pneumonia that required hospitalization and those that did not, the time course of changes in MDA levels was similar, except that an increase in MDA level was more pronounced in the hospitalized group on the 14th day (Figure 1B). Comparative analysis of 8-OHdG levels between hospitalized and outpatients with COVID-19 pneumonia has shown that its levels remained elevated in the hospitalized group between the 7th and 14th day, while at the same time, a drop in 8-OHdG was observed in outpatients (Figure 1D). What is more, although the similar AOPP profile was observed in both hospitalized and non-hospitalized patients with COVID-19 pneumonia, the values were markedly higher in hospitalized patients (*p* = 0.009), reaching the values from the admission on day 14 (Figure 1F).

### 3.2. The Correlation of Redox Biomarkers with Inflammatory Biomarkers and Multiorgan Impairment Biomarkers in Patients with COVID-19 Pneumonia at Different Time Points

The observed high levels of MDA and AOPP on admission lacked association with all indices of inflammation (Table 4), while observed correlations between 8-OHdG and ALT activity (rho = 0.30, *p* = 0.028) or creatinine concentration (rho = 0.39, *p* = 0.003) might suggest subclinical liver and/or kidney damage which contributes to systemic oxidative stress in the early phase of the disease (Table 5).

Seven days upon admission, a significant correlation of AOPP with inflammatory biomarkers, such as CRP (rho = 0.32, *p* = 0.016), ferritin (rho =0.32, *p* = 0.015) and absolute number of neutrophils (rho = 0.27, *p* = 0.041) was noted, while the correlation with multiorgan impairment biomarkers was not found (Table 5). Fourteen days upon admission, the levels of AOPP significantly correlated with IL−6 (rho = 0.28, *p* = 0.029), ferritin (rho = 0.26, *p* = 0.049), absolute number of monocytes (rho = 0.28, *p* = 0.037) and multiorgan impairment biomarkers such as the activity of plasma non-functional enzymes AST (rho = 0.44, *p* = 0.001) and LDH (rho = 0.29, *p* = 0.039), which might indicate supposed heart and/or erythrocyte damage (Table 4).

### 3.3. The Association of Redox Biomarkers with CT Pulmonary Patterns in COVID-19 Pneumonia

**Figure 2:**
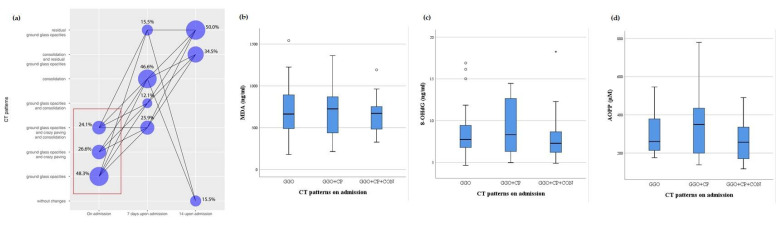
The frequency of CT patterns with their timely progression (**a**) and relation with the levels MDA (**b**), 8-OHdG (**c**), and AOPP (**d**) in COVID-19 patients on admission.; GGO-Ground Glass Opacities; CP-Crazy Paving; CON-Consolidation.

**Figure 3:**
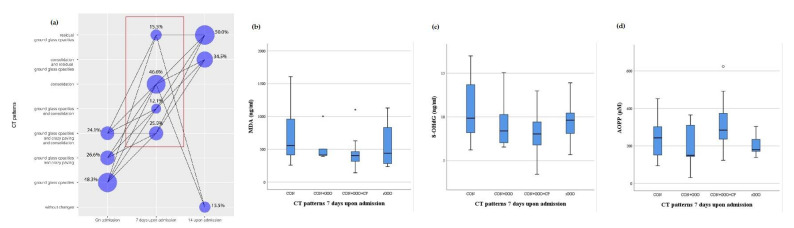
The frequency of CT patterns with their timely progression (**a**) and relation with the levels of MDA (**b**), 8-OHdG (**c**) and AOPP (**d**) in COVID-19 patients 7 days upon admission; GGO—ground glass opacities; CP—crazy paving; CON—consolidation; rGGO—residual ground glass opacities.

**Figure 4:**
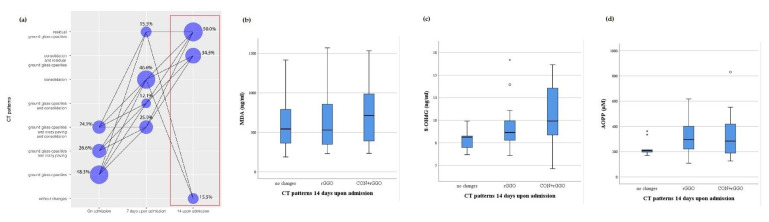
The frequency of CT patterns with their timely progression (**a**) and relation with the levels of MDA (**b**), 8-OHdG (**c**) and AOPP (**d**) in COVID-19 patients 14 days upon admission; CON—consolidation; rGGO—residual ground glass opacities.

On admission, the levels of redox biomarkers were assessed with regard to certain CT pulmonary patterns (Figure 2). Almost half of the patients had ground glass opacities (GGO) on admission (48.3%), while 7 days upon admission, consolidation (CON) was a dominant finding (46.6%) (Figure 2 and Figure 3). The association between MDA, 8-OHdG and AOPP values and CT pulmonary finding was lacking both on admission and 7 days upon admission (Figure 2 and Figure 3). Fourteen days after admission, the majority of patients had residual GGO (50%, Figure 4). At this time point, a certain yet insignificant trend was observed for MDA and 8OH-dG levels, with the lowest level of these biomarkers in patients without CT changes, as opposed to patients having the combination of CON and residual GGO (rGGO) (Figure 4). Similar to the pattern of changes in redox biomarkers, inflammatory biomarkers were higher in patients with more severe pulmonary CT findings (Figures S3–S5, provided).

## 4. Discussion

Our results on time-dependent relation of oxidative stress parameters with inflammatory and multiorgan impairment biomarkers, as well as pulmonary patterns in COVID-19 pneumonia patients, contribute to the ongoing challenge in understanding SARS-CoV2 infection mechanisms. In order to assess the sole effect of acute infection itself, along with the interplay of cytokine and free radical storm, COVID-19 patients having no co-morbidities, where the underlying mechanism would significantly contribute to oxidative stress, were recruited. Apart from observed changes in time course of redox biomarkers in patients with COVID-19 pneumonia, special emphasis should be placed on the fact that pattern of changes differs between non-hospitalized and hospitalized individuals.

Earlier data supported the association between oxidative stress, inflammation and the pathogenesis of severe acute respiratory syndrome coronavirus (SARS-CoV) infection that was caused by a previous viral strain [18]. Indeed, such data suggested that the onset of severe lung injury in SARS-CoV-infected patients depended on activation of the oxidative stress machinery that was coupled with innate immunity and activated transcription factors, such as NF- κB, resulting in an exacerbated pro-inflammatory host response [19]. As recently suggested by Polidori et al., the complexity of COVID-19 disease should not be regarded in line with the traditional medical paradigm ‘one cause—one mechanism—one therapy’ [13]. Indeed, apart from pneumonia and hypoxemia, biomolecular changes and multisystem impairment might represent a hallmark of the disease [13,20,21]. The association between inflammation and oxidative stress in infectious diseases is well established [22,23,24,25,26]. In this line, several reviews have suggested that redox homeostasis misbalance plays a part in COVID-19 pathophysiology, progression and outcome [1,2]. One of the significant underlying mechanisms in COVID-19 disease is the imbalance in redox homeostasis favoring pro-oxidant conditions, potentiated with inflammation-dependent oxidative stress [22,27,28]. The key non-enzymatic regulator of redox homeostasis is glutathione (GSH), which is, together with other thiol antioxidants, implicated in inflammation and host defense against infection [29]. Having in mind previous data which indicate that GSH deficiency in the alveolar fluid in acute respiratory distress syndrome (ARDS) augments lung cell injury caused by increased free radical production and inflammation, it seems reasonable that individual responsiveness to COVID-19 pneumonia might also be influenced by reduced GSH levels [9]. What is more, it has been proposed that GSH deficiency might be involved in both serious manifestations and even COVID-19 mortality [9]. In line with this finding are the results on erythrocyte GSH deficiency in COVID-19 patients when compared to controls [11]. On the contrary, there are data showing lack of any association between GSH levels and disease severity [14]. 

So far, malondialdehyde (MDA), as an established biomarker of lipid oxidative damage, was determined in COVID-19 patients to be stratified according to disease severity; however, this was without any association [11,14]. Similarly, in these patients, no association was observed for redox biomarkers of proteins, such as carbonyl content and sulphydryl levels [14]. However, a recent study of Kalem et al. potentiated the role of thiol-disulphide homeostasis as a novel indicator of intensity of oxidative stress [8]. Namely, both native and total thiols seem to have significant predictive value in COVID-19 diagnosis, as well as in estimation of disease severity [8]. Surprisingly, the data on biomarkers of oxidative damage to DNA in COVID-19 patients are lacking.

Since increased free radical production, as a hallmark of SARS-CoV2 infection, has the capacity to modify macromolecules over time, this cumulative oxidative macromolecular damage contributes to many mechanisms underlying COVID-19 disease progression. For that reason, it is of utmost importance not just to determine the level of oxidative stress parameters at one time point, but to evaluate their changes over time. Indeed, in our study, biomarkers of lipid, DNA and protein oxidative damage (MDA, 8-OHdG and AOPP, respectively) varied in three time points over a 14-day period. Namely, significant decrease was observed in MDA and AOPP levels in contrast to increase in 8-OHdG levels at the second time point (7 days upon admission). However, 14 days upon admission, a noticeable increase was observed both in MDA and AOPP levels. Advanced oxidation protein products comprise dityrosine, pentosidine and carbonyl-containing protein products. It is important to note that AOPP is a well-known biomarker of protein oxidative modifications which can be produced in the presence of hypochlorous acid (HOCl), a main product of neutrophil myeloperoxidase activity as a part of the innate response to pulmonary viral infections [30,31]. Namely, HOCl seems to contribute to multiorgan injury in COVID-19 by multiple mechanisms, such as competition with oxygen at heme binding sites, decrease in O_2_ saturation, hemoglobin-heme iron oxidation, heme destruction and iron release [32]. Thus our results on increased levels of neutrophils in a time-dependent manner suggest that changes in AOPP levels might be useful in clinical management of COVID-19 pneumonia patients. 

Furthermore, in some patients, a mild infection with upper respiratory tract symptoms might worsen and cause pneumonia by the end of the first week or the beginning of the second week. The so-called pulmonary phase can be divided into the pneumonia patient without hypoxia and those with hypoxia who will likely require hospitalization and oxygen supplementation. Therefore, in our study, a comparative analysis of redox biomarkers and possible multiorgan impairment has been determined in both hospitalized and non-hospitalized individuals with COVID-19 pneumonia. Indeed, although a similar pattern of changes was observed in all measured oxidative stress biomarkers, increase in MDA levels between the second and third time point was steeper in hospitalized patients in whom 8-OHdG remained elevated in this period.

In addition to pneumonia, evidence suggests that COVID-19 may affect other parts of the body as well, leading to cardiac injury and early renal damage [33]. This might be due to thrombosis, which is observed in small vessels of severely ill COVID-19 patients, suggesting alternative mechanisms for organ damage, such as hypoxemia and ischemia. 

In this process, hypoxemia-related cascades have the central role, associated with increased generation of reactive oxygen species [34,35]. In our cohort of patients, apart from relation with inflammation indices, a correlation between 8-OHdG and alanine aminotransferase activity, as well as creatinine concentration found at the second time point, might indicate early liver and/or kidney damage. Moreover, aspartate aminotransferase and lactate dehydrogenase, as potential multiorgan impairment biomarkers, were associated with AOPP levels 14 days upon admission, which might suggest heart and/or erythrocyte damage. What is more, the lowest levels of all three measured oxidative stress parameters were observed in patients in whom changes in CT pulmonary pattern were withdrawn.

There are certain limitations to this study, such as the relatively small number of patients. The effect of comorbidities was avoided by recruiting only subjects without diseases associated with marked oxidative stress, except for eight patients with regulated hypertension. Besides, the control group is lacking due to the obstacles in collecting blood samples from healthy individuals during the COVID-19 outbreak. However, we believe that all data clarifying the underlying role of oxidative stress in SARS-CoV-2 infection expand the possibilities of antioxidant therapy.

## 5. Conclusions

In the acute course of the COVID-19 disease, redox biomarkers correlate with certain inflammatory and multiorgan impairment biomarkers and are associated with changes in the chest MDCT findings in patients with COVID-19 pneumonia. Special emphasis should be placed on the fact that the pattern of changes differs between non-hospitalized and hospitalized individuals. Our results are in favor of the suggested involvement of neutrophils networks, IL-6 production, along with different organ/tissue involvement in systemic oxidative stress in COVID-19.

## Figures and Tables

**Table 1:** The characteristics of 58 patients with COVID-19.

Parameter	
Gender	
Male, n (%)	24 (41)
Female, n (%)	34 (59)
Age ^a^	48.20 ± 13.46
Systemic Arterial Hypertension, n (%)	8 (14)
BMI (kg/m^2^) ^a^	28.03 ± 5.34
Smoking, n (%) ^b^	1 (2)
Hospitalization	42 (72)

^a^ mean ± standard deviation; BMI—body mass index; ^b^ smokers were individuals who reported smoking every day during a minimum of 60-day period prior to their enrollment in this study.

**Table 2:** Administrated therapy in the cohort of COVID-19 patients over the course of the disease.

Therapy	On Admission	7 Days Upon Admission	14 Days Upon Admission
Oxygenation, n (%)	17 (29)	14 (24)	1 (2)
Vasoactive drugs, n (%)	8 (14)	8 (14)	8 (14)
Hydroxychloroquine n (%)	10 (17)	-	-
Low weight heparin, n (%)	40 (69)	40 (68)	17 (29)
Aspirin, n (%)	16 (28)	17 (29)	30 (52)
Tocilizumab, n (%)	-	1 (2)	-
Favipiravir, n (%)	15 (26)	-	-
Supplementation (Vitamin D, Vitamin C and Zn), n (%)	58 (100)	58 (100)	58 (100)

**Table 3:** Overview of the inflammatory biomarkers, multiorgan impairment biomarkers and redox biomarkers in 58 patients with COVID-19 pneumonia, on admission, 7 and 14 days upon admission.

Laboratory Parameters	On Admission	7 Days Upon Admission	14 Days Upon Admission	*p* Value
**Inflammatory Biomarkers**				
WBC (n)	5.0 ± 1.8	6.1 ± 1.9	6.6 ± 2.0	<0.001
Neutrophils (n)	3.0 ± 1.7	3.6 ± 1.8	3.8 ± 1.7	0.005
Lymphocytes (n)	1.4 ± 0.4	1.8 ± 0.5	1.4 ± 0.4	<0.001
Monocytes (n)	0.4 ± 0.2	0.5 ± 0.8	0.6 ± 0.2	<0.001
NLR	2.1 (0.4–8.9)	1.8 (0.8–24.6)	1.9 (0.9–8.5)	>0.05
IL-6 (pg/mL)	7.6 (1–112)	4 (1–45)	3 (1–41)	<0.001
CRP (mg/L)	7.2 (0.1–79.8)	3.9 (0.3–83.2)	1.8 (0.02–38.7)	<0.001
Ferritin (ng/mL)				
Female	80.5 (13.0–1500.9)	105.0 (13.0–612.0)	76.5 (12.0–536.0)	0.011
Male	350.0 (136.0–1500.9)	557.0 (156.0–1500.9)	401.0 (103.0–1500.9)	0.004
**Multiorgan impairment biomarkers**				
Urea (mmol/L)	4.5 ± 1.7	4.5 ± 2.3	5.3 ± 2.7	0.002
Creatinine (µmol/L)	85.1 ± 21.2	78.8 ± 16.0	83.8 ± 16.8	0.001
ALT (U/L)	33 (17–165)	59 (19–518)	53 (17–253)	0.002
AST (U/L)	22.5 (11–95)	35.5 (11–310)	26 (10–141)	0.018
LDH (U/L)	189.5 (99–473)	192.5 (86–581)	174 (91–411)	0.001
CK (U/L)	66.5 (12–565)	54.5 (15–809)	53.5 (19–518)	<0.001
**Redox biomarkers**				
MDA (ng/mL)	709.8 (178.4–1542.1)	477.5 (140.64–1583.6)	547.5 (230.6–1570.8)	0.002
8-OHdG (ng/mL)	7.7 (4.6–16.9)	8.9 (3.4–16.9)	8.6 (4.6–16.7)	0.006
AOPP (µM)	265.9 (117.3–779.67)	239.8 (31.3–623.8)	265.05 (108.2–830.3)	<0.001

WBC—white blood cells; NLR—neutrophil–lymphocytes ratio; CRP—C reactive protein; ALT—alanine aminotransferase; AST—aspartate aminotransferase; LDH—lactate dehydrogenase; CK—creatine kinase; MDA—malondialdehyde; 8-OHdG—8-hydroxy-2’-deoxyguanosine; AOPP—advanced oxidation protein products; depending on the type of variables and the normality of the distribution, results were presented as median (range) or mean± standard deviation. *p* for ANOVA with repeated measures or Friedman test.

**Table 4:** The correlation of redox biomarkers with inflammatory biomarkers in 58 patients with COVID-19 pneumonia on admission, 7 and 14 days upon admission.

On Admission	MDA (ng/mL) Rho	8-OHdG (ng/mL) Rho	AOPP (µM) Rho
IL 6 (pg/mL)	−0.08	−0.04	0.07
CRP (mg/L)	−0.13	−0.19	0.17
Ferritin (ng/mL)	−0.23	0.24	0.05
Lymphocytes (n)	0.18	0.03	−0.10
Neutrophils (n)	−0.06	0.08	−0.08
Monocytes (n)	0.08	−0.03	0.07
**7 days upon admission**			
IL 6 (pg/mL)	−0.10	−0.19	0.18
CRP (mg/L)	−0.18	−0.02	0.32 *
Ferritin (ng/mL)	−0.09	0.06	0.32 *
Lymphocytes (n)	0.22	0.16	−0.22
Neutrophils (n)	−0.06	−0.01	0.27 *
Monocytes (n)	0.23	0.18	0.04
**14 days upon admission**			
IL 6 (pg/mL)	0.02	0.19	0.28 *
CRP (mg/L)	−0.07	−0.01	0.21
Ferritin (ng/mL)	−0.19	0.01	0.26 *
Lymphocytes (n)	0.09	0.07	0.21
Neutrophils (n)	−0.21	0.01	0.18
Monocytes (n)	0.16	0.25	0.28 *

CRP—C reactive protein; MDA—malondialdehyde; 8-OHdG—8-hydroxy−2’-deoxyguanosine; AOPP—advanced oxidation protein products; rho—Spearman correlation coefficient; * *p* < 0.05.

**Table 5:** Correlation of redox biomarkers with multiorgan impairment biomarkers in 58 patients with COVID−19 pneumonia on admission, 7 and 14 days upon admission.

On Admission	MDA (ng/mL) Rho	8-OHdG (ng/mL) Rho	AOPP (µM) Rho
Urea (mmol/L)	−0.16	0.18	0.01
Creatinine (µmol/L)	−0.22	0.39 *	0.13
ALT (U/L)	−0.01	0.30 *	0.01
AST (U/L)	−0.05	0.23	0.09
LDH (U/L)	−0.10	0.01	0.01
CK (U/L)	−0.36	−0.01	0.17
**7 days upon admission**			
Urea (mmol/L)	−0.06	0.06	−0.03
Creatinine (µmol/L)	−0.09	0.02	0.15
ALT (U/L)	0.09	0.08	0.09
AST (U/L)	0.03	0.01	0.01
LDH (U/L)	−0.21	−0.19	0.01
CK (U/L)	−0.19	−0.05	−0.14
**14 days upon admission**			
Urea (mmol/L)	−0.18	0.05	0.01
Creatinine (µmol/L)	−0.09	0.20	0.25
ALT (U/L)	0.01	0.01	0.23
AST (U/L)	−0.02	−0.16	0.44 *
LDH (U/L)	−0.01	−0.15	0.29 *
CK (U/L)	−0.17	0.03	−0.14

ALT—alanine aminotransferase; AST—aspartate aminotransferase; LDH—lactate dehydrogenase; CK—creatine kinase; MDA—malondialdehyde; 8-OHdG—8-hydroxy-2’-deoxyguanosine; AOPP—advanced oxidation protein products; rho—Spearman correlation coefficient; * *p* < 0.05.

## Data Availability

The data supporting reported results can be found upon request in the form of datasets available at The University Hospital ’Dragiša Mišović-Dedinje’, Belgrade, Serbia and Institute of Medical and Clinical Biochemistry, Faculty of Medicine, University of Belgrade.

## Supplementary Materials

The following are available online at https://www.mdpi.com/article/10.3390/antiox10071126/s1, Figure S1: The temporal profiles of redox biomarkers (a) MDA, (b) 8-OHdG, (c) AOPP for each individual patient with COVID-19 pneumonia; MDA—malondialdehyde; 8-OHdG—8-hydroxy-2’-deoxyguanosine; AOPP—advanced oxidation protein products; Figure S2: The temporal profiles of redox biomarkers (a) MDA, (b) 8-OHdG, (c) AOPP for each hospitalized patient (red) and non-hospitalized patient (blue) with COVID-19 pneumonia; MDA—malondialdehyde; 8-OHdG—8-hydroxy-2’-deoxyguanosine; AOPP—advanced oxidation protein products; Figure S3. Time course of certain inflammatory parameters in relation to CT patterns on admission; GGO—ground glass opacities; CP—crazy paving; CON—consolidation; Figure S4. Time course of certain inflammatory parameters in relation to CT patterns 7 days upon admission; GGO—ground glass opacities; CP—crazy paving; CON—consolidation; rGGO—residual ground glass opacities; Figure S5. Time course of certain inflammatory parameters in relation to CT patterns 14 days upon admission; CON—consolidation; rGGO—residual ground glass opacities. Table S1. Overview of the inflammatory parameters, multiorgan impairment biomarkers and redox biomarkers in 42 hospitalized patients with COVID-19 pneumonia, on admission, 7 and 14 days upon admission, Table S2. Overview of the inflammatory parameters, multiorgan impairment biomarkers and redox biomarkers in 16 non-hospitalized patients with COVID-19 pneumonia, on admission, 7 and 14 days upon admission.
