# Supplementary material for: Time Course of Redox Biomarkers in COVID-19 Pneumonia: Relation with Inflammatory, Multiorgan Impairment Biomarkers and CT Findings
Source: Antioxidants (Basel). 2021 Jul 14;10(7):1126. doi: 10.3390/antiox10071126 (PMC8301049; doi:10.3390/antiox10071126)
Supplement: Supplementary file 1 [file antioxidants-10-01126-s001.zip › antioxidants-1264855-supplementary.pdf]

Supplementary Material: Time Course of Redox Biomarkers in COVID-19 Pneumonia: Relation with Inflammatory, Multiorgan Impairment Biomarkers and CT Findings

Tijana Kosanovic [1], Dragan Sagic [2], Vladimir Djukic [1], Marija Pljesa-Ercegovac [3], Ana Savic-Radojevic [3], Zoran Bukumiric [4], Miodrag Lalosevic [1], Marjana Djordjevic [1], Vesna Coric [3,*] and Tatjana Simic [3,5,*]

[1] Radiology Department, The University Hospital 'Dr. Dragisa Misovic- Dedinje', 11000 Belgrade, Serbia; tijana.kosanovic@dragisamisovic.bg.ac.rs (T.K.); office@dragisamisovic.bg.ac.rs (V.D.); miodrag.lalosevic@dragisamisovic.bg.ac.rs (M.L.); marjana.djordjevic@dragisamisovic.bg.ac.rs (M.D.)
[2] Institute for Cardiovascular Diseases "Dedinje", 11000 Belgrade, Serbia; dragan.sagic@med.bg.ac.rs (D.S.)
[3] Institute of medical and clinical biochemistry, Faculty of Medicine, University of Belgrade, 11000 Belgrade, Serbia; marija.pljesa-ercegovac@med.bg.ac.rs (M.P.-E.); ana.savic-radojevic@med.bg.ac.rs (A.S.R.)
[4] Institute of Medical Statistics and Informatics, University of Belgrade, School of Medicine, 11000 Belgrade, Serbia; zoran.bukumiric@med.bg.ac.rs (Z.B.)
[5] Serbian Academy of Science and Arts, 11000 Belgrade, Serbia
* Correspondence: vesna.coric@med.bg.ac.rs (V.C.); tatjana.simic@med.bg.ac.rs (T.S.); Tel.: +381-113643273 (V.C.); +381-113643250 (T.S.)

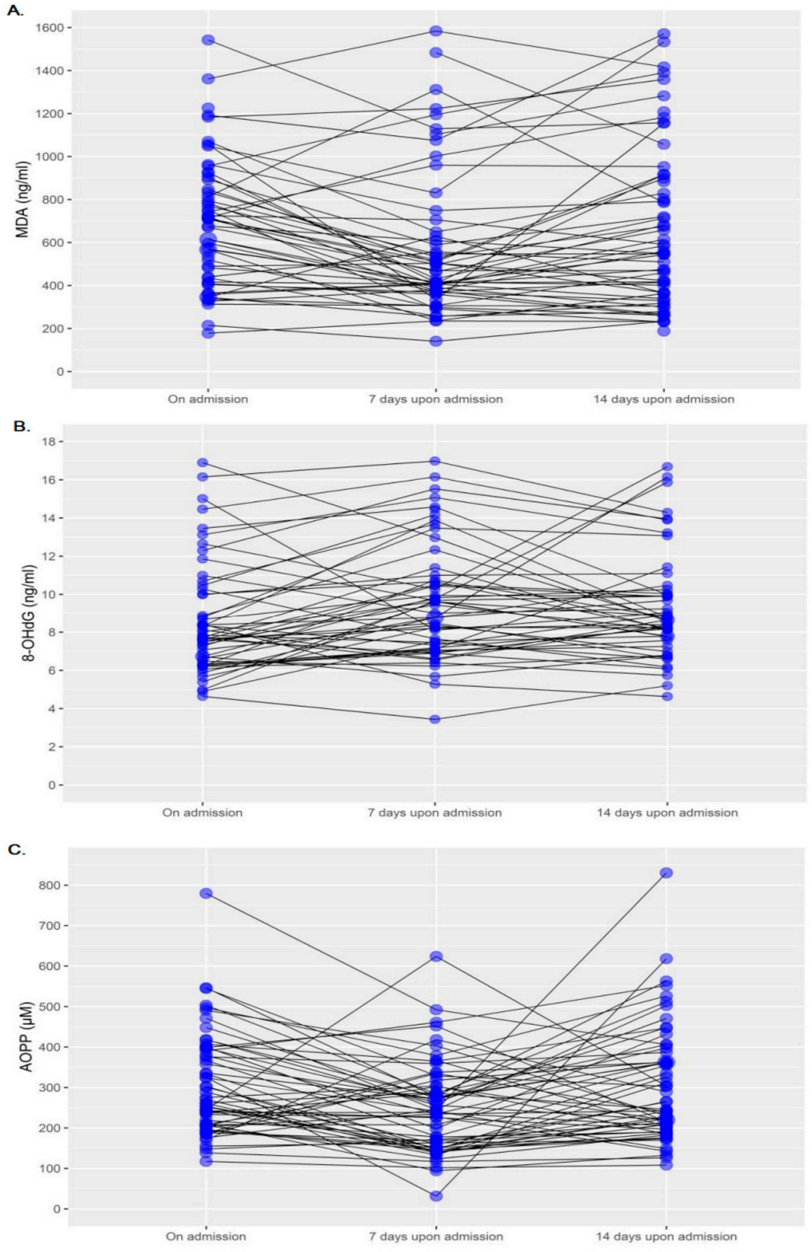

Figure S1. The temporal profiles of redox biomarkers **(a)** MDA, **(b)** 8-OHdG, **(c)** AOPP for each individual patient with COVID-19 pneumonia; MDA – malondialdehyde; 8-OHdG - 8-hydroxy-2'–deoxyguanosine; AOPP- Advanced oxidation protein products

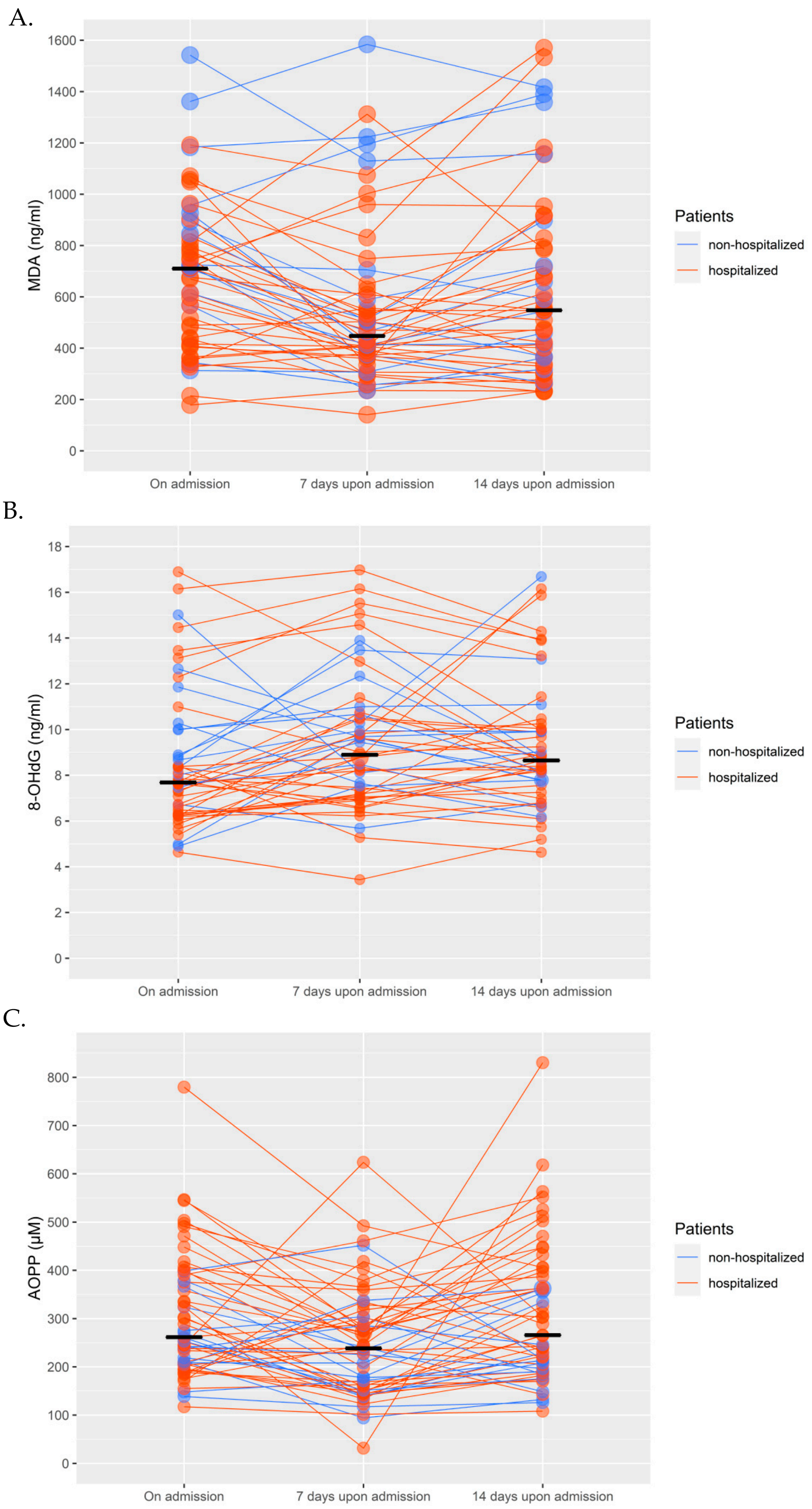

Figure S2: The temporal profiles of redox biomarkers **(a)** MDA, **(b)** 8-OHdG, **(c)** AOPP for each hospitalized patient (red) and non-hospitalized patient (blue) with COVID-19 pneumonia; MDA – malondialdehyde; 8-OHdG - 8-hydroxy-2'–deoxyguanosine;

AOPP- Advanced oxidation protein products

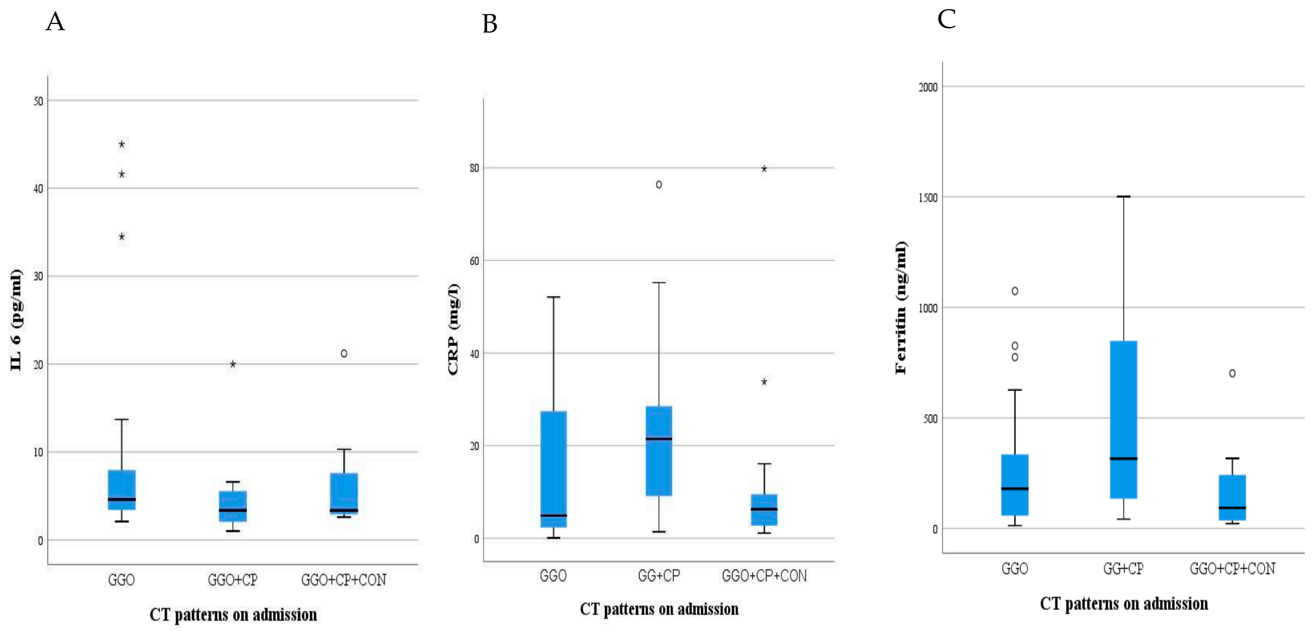

Figure S3. Time course of inflammatory parameters **(a)** IL-6, (**b**) CRP, (**c**) Ferritin in relation to CT patterns on admission; GGO-Ground Glass Opacities; CP-Crazy Paving; CON-Consolidation;

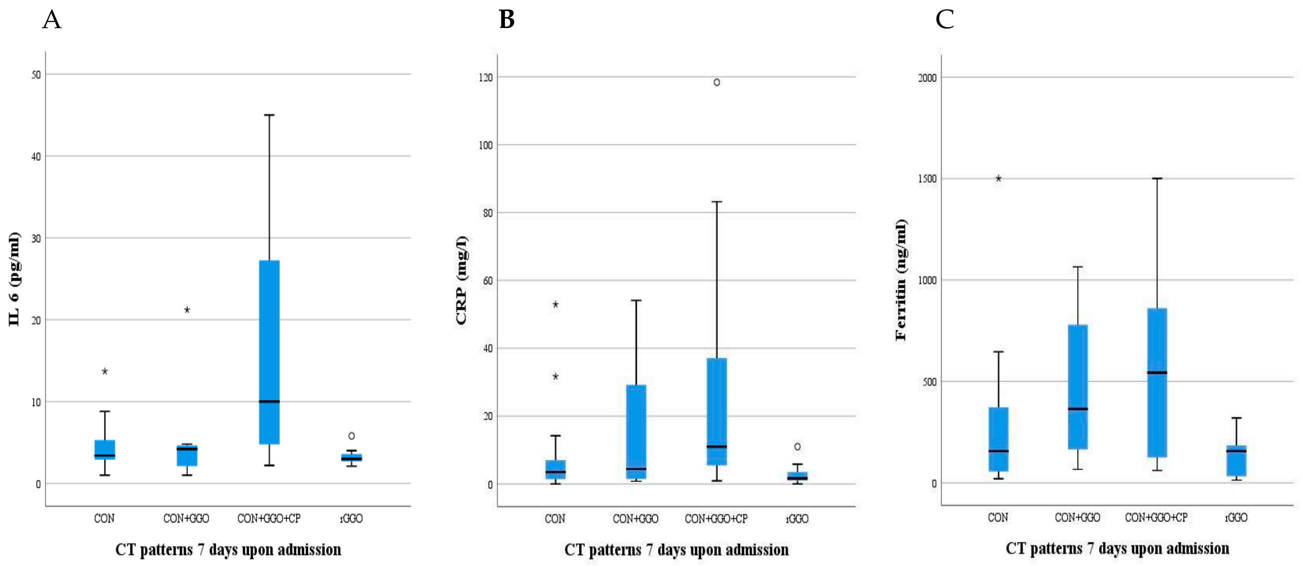

Figure S4. Time course of inflammatory parameters **(a)** IL-6, (**b**) CRP, (**c**) Ferritin in relation to CT patterns 7 days upon admission; GGO-Ground Glass Opacities; CP-Crazy Paving; CON-Consolidation; rGGO- residual Ground Glass Opacities;

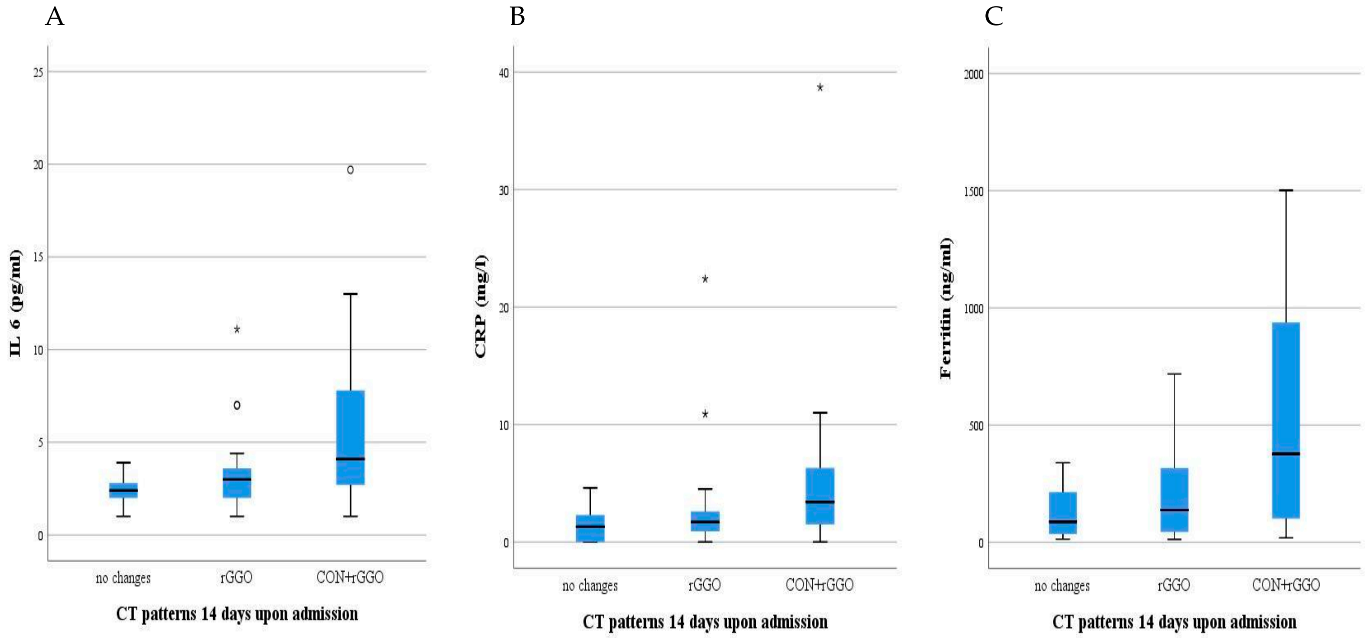

Figure S5. Time course of inflammatory parameters **(a)** IL-6, (**b**) CRP, (**c**) Ferritin in relation to CT patterns 14 days upon admission; CON-Consolidation; rGGO- residual Ground Glass Opacities;

Table S1. Overview of the inflammatory parameters, multiorgan impairment biomarkers and redox biomarkers in 42 hospitalized patients with COVID-19 pneumonia, on admission, 7 and 14 days upon admission.

Laboratory parameters	On admission	7 days upon admission	14 days upon admission	P value
**Inflammatory parameters**				
WBC (n)	5.2±1.9	6.1±1.9	6.9±2.1	<0.001
Neutrophils (n)	3.2±1.8	3.6±1.9	4.0±1.8	0.018
Lymphocytes (n)	1.4±0.4	1.7±0.6	2.0±0.6	<0.001
Monocytes(n)	0.5±0.2	0.5±0.2	0.6±0.2	<0.001
NLR	2.3 (0.4-8.9)	1.7 (0.8-24.6)	1.9 (0.5-8.5)	0.395
IL-6 (pg/ml)	11.1 (2.1-112.0)	4.5 (1.0-45.0)	3.1 (1.0-41.0)	<0.001
CRP (mg/l)	15.5 (1.1-79.8)	5.2 (0-83.2)	2.3 (0-38.7)	<0.001
Ferritin (ng/ml)				
Female	89.0 (13.0-1005.9)	124.5 (20.0-612.0)	87.5 (12.0-536.0)	0.030
Male	378.0 (136.0-1500.9)	617.5 (218.0-1500.9)	531.0 (103.0-1500.9)	0.001
**Multiorgan impairment biomarkers**				
Urea (mmol/L)	4.7±1.8	4.5±2.7	5.6±3.0	<0.001
Creatinine (µmol/L)	86.4±22.7	78.0±16.4	84.8±17.2	0.004
ALT (U/L)	33.5 (17-165)	66.5 (20-518)	61 (20-253)	<0.001
AST (U/L)	25 (13-95)	42 (15-310)	29.5 (11-141)	0.001
LDH (U/L)	198.5 (120-473)	199 (129-581)	180 (91-411)	0.007
CK (U/L)	70 (12-180)	52 (15-809)	52 (19-518)	0.136
**Redox biomarkers**				
MDA (ng/ml)	614.2 (178.4-1191.8)	416.7 (140.6-1311.6)	546.6 (230.6-1570.9)	0.057
8-OHdG (ng/ml)	7.5 (4.6-16.9)	8.6 (3.4-16.9)	8.7 (4.6-16.1)	0.013
AOPP (µM)	301.7 (117.3-779.7)	259.3 (31.3-623.8)	307.8 (108.2-830.3)	0.001

WBC- white blood cells; NLR- neutrophil-lymphocytes ratio; CRP- C reactive protein; ALT- alanine aminotransferase; AST- aspartate aminotransferase; LDH- lactate dehydrogenase; CK- creatine kinase; MDA – malondialdehyde; 8-OHdG - 8-hydroxy-2' –deoxyguanosine; AOPP- Advanced oxidation protein products; Depending on the type of variables and the normality of the distribution, results was presented median (range) or mean± standard deviation. p for ANOVA with repeated measures or Friedman test;.

Table S2. Overview of the inflammatory parameters, multiorgan impairment biomarkers and redox biomarkers in 16 non-hospitalized patients with COVID-19 pneumonia, on admission, 7 and 14 days upon admission.

Laboratory parameters	On admission	7 days upon admission	14 days upon admission	P value
**Inflammatory parameters**				
WBC (n)	4.7±1,3	6.1±1.9	5.7±1.5	0.047
Neutrophils (n)	2.6±0.9	3.5±1.6	3.3±1.2	0.028
Lymphocytes (n)	1.5±0.5	1.9±0.5	1.8±0.5	0.024
Monocytes(n)	0.4±0.2	0.5±0.1	0.5±0.1	0.222
NLR	1.8 (0.4-3.1)	1.9 (0.8-3.7)	1.9 (0.6-3.1)	0.305
IL-6 (pg/ml)	3.5 (1.0-22.8)	2.9 (1.0-5.8)	2.4 (1.0-7.0)	0.043
CRP (mg/l)	2.9 (0.1-52.1)	1.5 (0-11.0)	1.1 (0-10.9)	0.004
Ferritin (ng/ml)				
Female	39.0 (17.0-242.0)	34.0 (13.0-184.0)	38.0 (17.0-150.0)	0.255
Male	285.5 (221.0-775.0)	295.0 (156.0-646.0)	308.5 (137.0-491.0)	0.223
**Multiorgan impairment biomarkers**				
Urea (mmol/L)	4.1±1.1	4.5±1.2	4.6±1.3	0.345
Creatinine (µmol/L)	82±16.9	80.9±15.5	80.9±15.9	0.834
ALT (U/L)	32 (22-66)	42.5 (19-81)	38 (17-123)	0.984
AST (U/L)	20 (11-38)	24 (11-47)	22.5 (10-61)	0.713
LDH (U/L)	176.5 (99-306)	160.5 (86-232)	158 (96-208)	0.021
CK (U/L)	64.5 (25-565)	47 (29-599)	54.5 (39-474)	0.611
**Redox biomarkers**				
MDA (ng/ml)	724.9 (312.9-1542.2)	504.9 (235.8-1583.6)	591.3 (265.8-1416.7)	0.022
8-OHdG (ng/ml)	8.7 (4.9-15.0)	9.7 (5.7-13.9)	8.4 (6.2-16.7)	0.269
AOPP (µM)	248.1 (138.6-398.8)	193.6 (94.2-452.0)	210.8 (125.9-362.2)	0.068

WBC- white blood cells; NLR- neutrophil-lymphocytes ratio; CRP- C reactive protein; ALT- alanine aminotransferase; AST- aspartate aminotransferase; LDH- lactate dehydrogenase; CK- creatine kinase; MDA – malondialdehyde; 8-OHdG - 8-hydroxy-2' –deoxyguanosine; AOPP- Advanced oxidation protein products; Depending on the type of variables and the normality of the distribution, results was presented median (range) or mean± standard deviation. p for ANOVA with repeated measures or Friedman test;.
